# Heavy Metals in the Soils of Placer Small-Scale Gold Mining Sites in Myanmar

**DOI:** 10.5696/2156-9614-10.27.200911

**Published:** 2020-08-25

**Authors:** Aung Zaw Tun, Pokkate Wongsasuluk, Wattasit Siriwong

**Affiliations:** 1 International Postgraduate Program in Hazardous Substance and Environmental Management, Chulalongkorn University, Bangkok, Thailand; 2 Center of Excellence on Hazardous Substance Management, Chulalongkorn University, Bangkok, Thailand; 3 College of Public Health Science, Chulalongkorn University, Bangkok, Thailand; 4 Health and Social Sciences and Addiction Research Unit (HSSRU), Chulalongkorn University, Bangkok, Thailand

**Keywords:** heavy metals, gold mining, soil, Myanmar

## Abstract

**Background.:**

Artisanal and small-scale mining activities are widely practiced globally. Concentrations of heavy metals associated with gold, such as copper (Cu), zinc (Zn), arsenic (As), cadmium (Cd), mercury (Hg) and lead (Pb) can increase in the environment as a result of mining activities, leading to environmental pollution and pose toxicity risks to humans and animals.

**Objectives.:**

The aim of the present study was to investigate soil concentrations of toxic heavy metals in placer small-scale gold mining operations in Myanmar.

**Methods.:**

Soil samples were collected from three placer small-scale gold mining sites: Site A located in the Hmawbon public protected forest, Site B and Site C, situated in the Nant-Kyin reserved forest around Nar Nant Htun village. At each site, soil samples were collected from four gold mining stages (ore processing, sluicing, panning, and amalgamation). Atomic absorption spectroscopy was utilized to examine the concentrations of As, Cd, Pb, and Hg.

**Results.:**

The highest heavy metal concentrations were generally found in the amalgamation stages across all the gold mining sites. Across the three mining sites, the maximum heavy metal concentrations in the amalgamation stage were 22.170 mg.kg^−1^ for As, 3.070 mg.kg^−1^ for Cd, 77.440 mg.kg^−1^ for Hg, and 210.000 mg.kg^−1^ for Pb.

**Conclusions.:**

The present study examined the concentrations of As, Cd, Hg and Pb in the soil of several small-scale gold mining sites in Banmauk Township, Myanmar. The results demonstrated the presence of high concentrations of heavy metals in the soil of the gold mining sites. Miners in this area work without proper personal protective equipment, and frequent exposure to heavy metals in the soil may cause adverse health effects. The present study provides baseline data for future risk assessment studies of heavy metal contamination in gold mines.

**Competing Interests.:**

The authors declare no competing financial interests

## Introduction

Artisanal and small-scale mining activities are widely practiced across the globe, from Central and South America to Africa, and Oceania to Asia.^1^ Around 3 to 5 times more income is generated from mining activities than from small-scale forestry, fisheries and agriculture in many countries. Artisanal and small-scale mining is conducted around the world by approximately 20 to 30 million people and gold mining is the most common form of artisanal and small-scale mining in several countries.^2^

However, concentrations of heavy metals associated with gold, such as copper (Cu), zinc (Zn), arsenic (As), cadmium (Cd), mercury (Hg) and lead (Pb) can increase in the environment through gold mining activities, resulting in pollution in the environment and toxicity to humans and animals.^3^–^7^ Based on the carcinogenicity and likelihood of occurrence, the most toxic heavy metals to animals and humans are As, Cd, Hg and Pb.^8^–^9^ Furthermore, As, Cd, Hg and Pb have high toxicity due to their elemental impurities, long life and persistence in soil. These heavy metals are easily dispersed and can accumulate in plants and animals and bioaccumulate in humans through the food chain. Moreover, they can cause adverse health effects in humans via carcinogenic and non-carcinogenic effects even at exposure to low concentrations.^10^–^12^ In addition, Tóth *et al.* reported that mining caused increasing As, Cd, Hg and Pb concentrations in surrounding soils of mining areas in Europe.^13^ A substantial relationship between As concentrations in the environment and gold mining activities has been reported by Harmanescu *et al.* and Silva *et al.*^14^–^15^ In Ghana, Ahmad and Carboo reported a maximum As concentration of 8305 mg.kg^−1^ and Bempah *et al.* found a maximum As concentration of 1752 mg.kg^−1^ in gold mine tailings.^16^–^17^ In Tanzania, 6- to 11-fold higher concentrations of Cd have been reported compared to uncontaminated soil (1 mg.kg^−1^).^18^ Telmer and Veiga estimated that about 640–1350 mg of Hg are released annually into the environment due to mining activities.^19^ Therefore, mining is the main anthropogenic source of Hg.^19^–^20^ High Hg concentrations in the soil of gold mining areas have also been reported in Iran and Kenya of 100 mg.kg^−1^ and 1920 mg.kg^−1^, respectively^20^–^21^ In addition, 80 mg.kg^−1^ and 510 mg.kg^−1^ of Pb concentrations in gold mining soils have been reported in Oman and Kenya, respectively^22^–^23^

In Myanmar, a developing country, gold mining activities are concentrated in four regions: Bago, Sagaing, Mandalay and Kachin.^24^ In these regions, placer deposits are widespread, and gold is commonly extracted through the placer mining technique. Small-scale gold mining plays an important role in Myanmar's gold production. The CEIC database reported that gold production in Myanmar was 1692 kg in 2015 and 1700 kg in 2016.^25^ However, there have been few studies of the impacts of gold mining in Myanmar. The aim of the present study was to investigate As, Cd, Hg and Pb concentrations in the soil of placer small-scale gold mining stages in Banmauk Township, Sagaing Region. The resulting data on heavy metal contamination in the soil of Myanmar gold mining sites can serve as a baseline for future studies.

**Abbreviations**
ASGM: Artisanal and small-scale gold mining

## Methods

**Figure 1:**
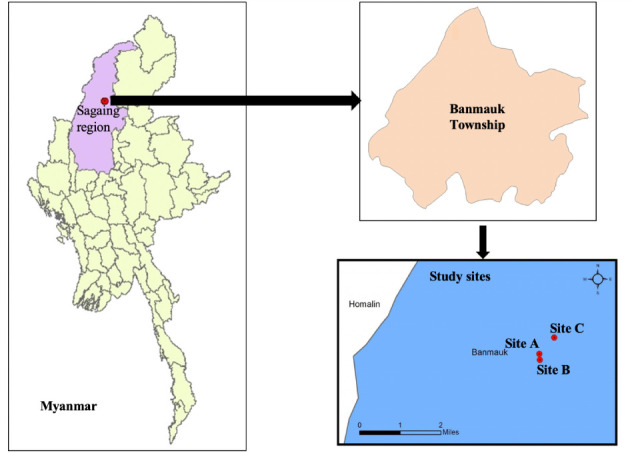
Mining sites located in Banmauk Township, Sagaing Region, Myanmar

The present study was conducted in Banmauk Township, a major placer gold mining area in the Sagaing Region of Myanmar. In Banmauk Township, soil samples were collected in three placer small-scale gold mining sites: Site A located in the Hmawbon public protected forest, and Site B and Site C situated in the Nant-Kyin reserved forest around Nar Nant Htun village, which is located about 46.7 km north-east of Banmauk Town *(Figure 1).* In Nar Nant Htun village, there are around 120 households with a population of 400, and most of the inhabitants of this village work as miners, farmers, and shifting cultivators (taungya). Placer deposits, from which gold can be easily accessed and extracted, are widely found in the vicinity of this village.^24^

**Figure 2:**
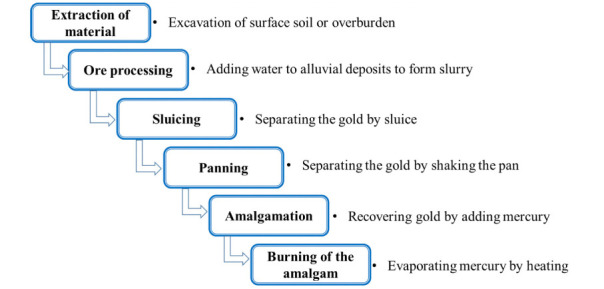
Placer small-scale gold mining processes

In Myanmar, placer gold mining is generally carried out in 6 stages as shown in Figure 2.

**Figure 3:**
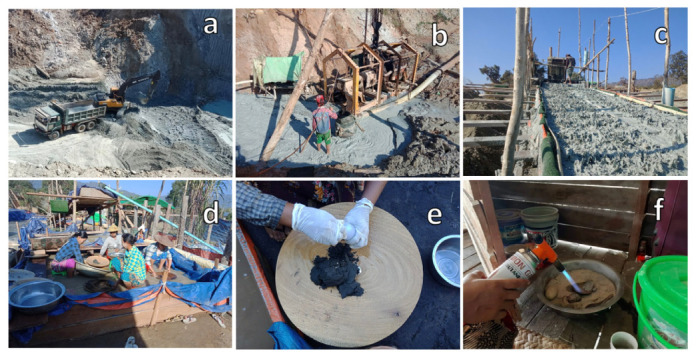
Placer small-scale gold mining stages in Banmauk Township: (a) excavation of surface soil through backhoe and truck; (b) ore processing by adding water; (c) sluicing through a wooden sluice; (d) panning with wooden pan; (e) recovering gold particles by adding Hg; (f) heating in order to evaporate Hg and recover gold. January 25, 2019. (Photos by author)

For the material extraction, the topsoil over the placer deposits (ore) is removed and ore is mined using backhoes and dump trucks *(Figure 3a).* Next, the extracted ore is processed to obtain slurry by mixing water into the placer deposits to liberate gold from other minerals in the ore processing *(Figure 3b).* Once the gold is liberated, it is then concentrated into a smaller mass through gravity methods of sluicing and panning. In the sluicing stage, gold-containing soil is caught by waterproof carpets from the liberated ore slurry through sluicing *(Figure 3c).*

The gold-containing soil attained from the sluicing stage is then concentrated again by hand-shaking the pan in the panning stage *(Figure 3d).* Next, Hg is added into the smaller amount of the gold-containing soil attained from the panning stage to capture the liberated gold particles *(Figure 3e).* Mercury then adheres to the gold particle surface, because the surface tension of gold is higher than that of Hg. In order to attain amalgam, the Hg and gold particle mixture is then placed into a fabric bag and squeezed by hand and the soil is removed. In some mining sites, this Hg-rich soil residue is then reused about 3–5 times for further recovery of gold through cyanide leaching techniques. After obtaining the amalgam, it is heated in order to evaporate Hg and to recover gold *(Figure 3f).*^26^–^27^

The material extraction stage requires machines such as backhoes and dump trucks to extract and carry the ore in place of manual labor. In the other gold mining stages, manual labor is required. The burning of the amalgam is normally conducted in a specific place or room. In this stage, laborers are exposed to Hg vapor via inhalation. The ore processing, sluicing, panning and amalgamation stages require manual labor, and these stages have a higher potential for occupational exposure to heavy metals. Therefore, the present study focused on the ore processing, sluicing, panning, and amalgamation stages of small-scale gold mining process in Myanmar.

### Soil sample collection

Three soil samples from each gold mining stage of ore processing, sluicing, panning, and amalgamation were collected from the surface to a 10 cm depth from three gold mining sites. In order to obtain a representative soil sample from each sampling point, five (5) replicated samples were gathered within a 2 m x 2 m grid through a steel trowel, carefully mixed up and then 50 g of soil sample from the mixed soil sample were collected.^28^ The collected homogenous soil samples were then placed into pre-washed polyethylene plastic bags with 10% nitric acid and distilled water and stored in an ice box.^29^–^30^ As soon as the samples were collected, they were sent to the Pesticide Analytical Laboratory under the Department of Agriculture, Ministry of Agriculture, Livestock and Irrigation, Myanmar in order to determine the concentrations of As, Cd, Hg, Pb and soil pH.

### Soil sample analysis

In the laboratory, soil samples were air dried for 2–3 days and sieved through a 2-mm nylon mesh to obtain a homogenous sample. Next, 5 g dry weight of each fine powder soil sample was mixed with 21 ml concentrated hydrochloric acid in a 250 ml beaker and swirled. The mixture was then swirled to mix well after adding 7 ml concentrated nitric acid. For the heavy metal dissolution, the soil samples were then digested on a hot plate at 115° for about 1–2 hours in a fume cupboard. The digested soil samples were then filtered using Whatman No. 40 filter paper after allowing them to cool for about 20 minutes. Next, hot distilled water was used to wash the filtrates. The filtrates were then collected and made up to volume with distilled water in a 100 ml volumetric flask. Finally, that volume was applied to determine the heavy metal concentrations through atomic absorption spectroscopy. An Agilent Technologies 200 Series 240FS AA model was applied to analyze the concentrations of Pb and Cd and the concentrations of As and Hg were determined using Agilent 240FS AA with Graphite Tube Atomizer 120 and Agilent 240FS AA with Agilent Vapor Generation Accessory (VGA 77), respectively. In addition, a Thermo Scientific Orion Star A211 pH Benchtop Meter was used to determine the soil pH in the ratio of 1:2.5 for soil and water. After the pH determination of 10 soil samples, a pH meter calibration was carried out through pH buffers at 4, 7 and 10.

### Quality control

For quality control, the laboratory followed the Association of Analytical Community (AOAC) Peer-Verified Methods Program.^31^ AnalaR grade reagents that meet with the standards of the American Chemical Society Committee on Analytical Reagents were used for digestion of soil samples. Atomic absorption spectroscopy carried out with auto calibration for quantification and the R^2^ was greater than or equal to 0.999. An auto sampler introduced the digested samples into the atomic absorption spectroscopy with a wavelength of 193.3 nm. The limit of detection was 0.0007 mg.kg^−1^ for As, 0.0099 mg.kg^−1^ for Cd, 0.0003 mg.kg^−1^ for Hg and 0.069 mg.kg^−1^ for Pb, and the limit of quantification was 0.0022 mg.kg^−1^ for As, 0.033 mg.kg^−1^ for Cd, 0.001 mg.kg^−1^ for Hg and 0.232 mg.kg^−1^ for Pb. Each sample was analyzed in duplicate, and before analyzing each heavy metal, blank solutions were analyzed to account for any contamination through the acids used in the digestion process. Relative standard deviations of the heavy metals were less than 5%.

## Results

Four heavy metal soil concentrations were investigated: As, Cd, Hg and Pb. Table 1 presents the heavy metal concentrations and soil pH level for each placer gold mining stage. Significant differences in heavy metal concentrations across the different gold mining stages in each site were identified through an independent t-test analysis.

**Table 1:** Heavy Metal Concentrations and Soil pH Levels

**Gold mining stages**	**As** **mean ± SD (mg.kg^−1^)**	**Cd** **mean ± SD (mg.kg^−1^)**	**Hg** **mean ± SD (mg.kg^−1^)**	**Pb** **mean ± SD (mg.kg^−1^)**	**pH** **mean ± SD**
Ore processing					
Site A	5.69 ±0.14	0.27 ±0.01	0.47 ± 0.23	9.73 ± 0.64	7.43 ± 0.42
Site B	6.55±1.25	0.27±0.02	0.68±0.43	11.80±1.73	5.91 ±0.35
Site C	1.80±0,48	0.33±0.04	0.53±0.13	12.80±2.99	8.57 ±0.11
Average ± SD	**4.68 ±2.53**	**0.29 ±0.03**	**0.56 ±0.11**	**11.44 ±1.57**	**7.30 ±1.33**
Sluicing					
Site A	1.04±0.19	0.36±0.02	0.15±0.03	8.60±1.56	8.54 ±0.22
Site B	9.02±5.60	0.40±0.12	0.35±0.11	10.27±3.50	6.91 ±0.58
Site C	1.31±0.22	0.13±0.06	0.51±0.04	7.67±0.90	8.45 ±0.10
Average ± SD	**3.79 ±4.53**	**0.30 ±0.15**	**0.34 ±0.18**	**8.85 ±1.32**	**7.97 ±0.92**
Panning					
Site A	1.24±0.42	1.32±0.16	4.86±5.87	34.40±13.22	7.73 ±0.15
Site B	16.25±2.29	1.67±0.89	1.53±0.49	74.67±65.62	7.70 ±0.18
Site C	14.89±6.97	0.61±0.36	0.58±0.31	27.33±4.16	7.95 ±0.50
Average ± SD	**10.79±8.30**	**1.20 ±0.54**	**2.32 ±2.25**	**45.47±25.54**	**7.79 ±0.14**
Amalgamation					
Site A	15.58±11.98	3.03±0.47	40.95±5.81	75.67±25.01	4.87 ±2.12
Site B	20.72±7.38	3.07±0.35	77.44±16.73	210.00±17.32	7.78 ±0.10
Site C	22.17±12.58	1.13±0.75	35.73±1.88	39.33±7.51	7.39 ±0.06
Average ± SD	**19.49±3.46**	**2.41 ±1.11**	**51.37±22.72**	**108.33±89.90**	**6.68 ±1.58**

At site A, As soil concentrations decreased in the order of amalgamation, ore processing, and panning followed by sluicing. For Cd, the order decreased across the following stages: amalgamation, panning, sluicing and ore processing. For Hg and Pb, at site A, the concentrations were highest during the amalgamation stages followed by panning, ore processing and lowest in the sluicing stage. However, according to the t-test results, there were no significant differences in heavy metal concentrations across gold mining stages. For As, significantly different concentrations were found between the ore processing and sluicing stage (p = 4.589 × 10-6 < 0.05) and the ore processing and panning stage (p = 6.385 × 10-5 < 0.05). For Cd, significantly different concentrations were found between the ore processing and sluicing stage (p = 0.002), ore processing and panning stage (p = 0.0003), ore processing and amalgamation stage (p = 0.005), sluicing and panning stage (p = 0.0004), sluicing and amalgamation stage (p = 0.0006), and the panning and amalgamation stage (p = 0.004). For Hg, significantly different concentrations were found between the amalgamation and ore processing stage (p = 0.0003), amalgamation and sluicing stage (p = 0.0002), and between the amalgamation and panning stage (p = 0.001). For Pb, significant differences were observed between the ore processing and panning stage (p = 0.03), ore processing and amalgamation stage (p = 0.01), sluicing and panning stage (p value = 0.03) and the sluicing and amalgamation stage (p value = 0.01).

In site B, As and Cd concentrations declined in the following stages: amalgamation, panning, sluicing, and ore processing. For Hg and Pb, concentrations were highest in the amalgamation stage, decreased in the panning and ore processing stages and lowest in the sluicing stage. For As, there were significant differences between the ore processing and panning stage (p = 0.003) and the ore processing and amalgamation stage (p = 0.03). Significantly different Cd concentrations were found between the ore processing and amalgamation stage (p = 0.0001) and between the sluicing and amalgamation stage (p = 0.0002). For Hg, significant differences were found between the amalgamation and ore processing stage (p = 0.001), amalgamation and sluicing stage (p = 0.001), amalgamation and panning stage (p = 0.001), and sluicing and panning stage (p = 0.01). For Pb, significant differences were found between the amalgamation and ore processing stage (p = 3.90 × 10-5), amalgamation and sluicing stage (p = 4.01 × 10-5) and between the amalgamation and panning stage (p = 0.03).

In site C, heavy metal concentrations decreased in the following order: amalgamation, panning, ore processing and sluicing. For As, significant differences were found between ore processing and panning (p = 0.03), sluicing and panning (p = 0.03), amalgamation and ore processing (p = 0.04) and amalgamation and sluicing (p = 0.04). For Cd, significant differences were found between ore processing and sluicing only (p = 0.01). For Hg concentrations, significant differences were found between the amalgamation and ore processing stage (p = 5.47 × 10-6), amalgamation and sluicing (p = 5.41 × 10-6) and amalgamation and panning (p = 5.75 × 10-6). For Pb, significant differences were found between ore processing and sluicing (p = 0.04), ore processing and panning (p = 0.007), ore processing and amalgamation (p = 0.005), sluicing and panning (p = 0.001) and between sluicing and amalgamation (p = 0.002).

The soil pH level ranged from 4.87–8.54 in Site A, 5.91–7.78 in Site B; and 7.39–8.57 in Site C.

## Discussion

For the mining sites in the present study, the highest concentrations of heavy metals generally occurred in the amalgamation stage. The gold recovery method in the placer gold mining process is the biggest contributing factor to these results. In this process, gravity methods of sluicing and panning were used to attain large amounts of gold in the amalgamation stage as gold has a greater density (19.3 g/cm^−3^) than other minerals in the ore. Likewise, the densities of As, Cd, Hg and Pb are 5.7 g.cm^−3^, 8.7 g.cm^−3^, 13.6 g.cm^−3^ and 11.34 g.cm^−3^, respectively, greater than the other minerals in the ore.^32^–^36^ Thus, relatively higher concentrations of these heavy metals were found in the amalgamation stage, along with gold.

According to the t-test results, Hg concentrations in the soil in the amalgamation stage were significantly greater than in the other gold mining stages at all of the gold mining sites. In addition, Hg can be directly released into the nearby sediment, soil and water, as Hg is mainly applied to extract gold from the ore through forming a stable amalgam. Furthermore, Hg can also be released into the atmosphere due to the burning of the amalgam in order to recover gold by evaporating Hg. As a consequence, it can pollute the atmosphere, rivers, lakes, underground water and soil, and even cause bio-concentration of methyl-mercury due to the methylation process via bacteria and the food chain, leading to serious health problems and environmental issues. Kawakami *et al.* reported a maximum Hg concentration in the atmosphere around a gold mining area in Thabeikkyin Township, Mandalay Division of Myanmar of 74 000 ng.m^−3^ and average Hg concentrations in hair of 1.5 μg.g^−1^ for artisanal and small-scale gold mining (ASGM) workers and 1.1 μg.g^−1^ for non-mineworkers, and Hg concentrations in groundwater pumped from a gold mining tunnel and the Ayeyarwaddy River in the ASGM area of 4.5 ng.L^−1^ and 29–35 ng.L^−1^, respectively.^37^ In addition, Osawa and Hatsukawa reported a Hg concentration in a sediment sample of the Ayeyarwaddy River in the ASGM area of the Mandalay and Sagaing regions of 81 μg.g^−1^, and average Hg concentrations in the hair of ASGM workers and non-mineworkers of 2.9 μg.g^−1^ and 1.2 μg.g^−1^, respectively.^24^

In the present study, the highest As concentration of 22.17 mg.kg^−1^ was found in Site C. This concentration was radically lower than the concentrations in the soil of the gold mining areas of previous studies in Ghana (8305 mg.kg^−1^), (1752 mg.kg^−1^), Portugal (820 mg.kg^−1^) and South Africa (79.40 mg.kg^−1^).^14^, ^16^–^17^, ^38^ For Cd, the highest concentration was found in Site B at 3.07 mg.kg^−1^, which was lower than the concentration in previous studies in Tanzania (6.40–11.70 mg.kg^−1^) and higher than reported in South Africa (0.05 mg.kg^−1^).^18^, ^38^ The highest Hg concentration found in Site B (77.44 mg.kg^−1^) was drastically lower than the concentrations of previous studies in Kenya (1920 mg.kg^−1^) and Iran (100 mg.kg^−1^), and higher than that of a study in South Africa (0.090 mg.kg^−1^).^20^–^21^, ^38^ In the case of Pb, 210 mg.kg^−1^ was reported to be the highest concentration at Site B. The highest concentration of Pb in the present study was 42 times greater than the concentration of a previous study in South Africa (4.790 mg.kg^−1^) and approximately 3 times higher than the concentration in a study in Oman (80 mg.kg^−1^).^22^, ^38^ However, the highest Pb concentration in this study was approximately 2 times lower than that of a previous study in Kenya (510 mg.kg^−1^).^23^

In the present study, soil pH ranged from very strongly acidic at 4.87 to strongly alkaline at 8.57, and most of the soil samples were found to be alkaline. In a previous study in a gold mining area north of Atbara, Sudan, the soil pH level was described as alkaline with a range of 7.46–8.8.^39^ However, strong acidity was found in the amalgamation stage of Site A in this study with a pH of 4.87. This could be a result of the further gold recovery from mercury-rich soil through the cyanide leaching method in Site A. In the cyanide leaching method, sodium cyanide is added into the mercury-rich soil in order to solubilize the gold, thereby recovering gold through active carbon that can absorb metal onto its surface from the solution of gold-cyanide complexes.^40^–^41^ Afterwards, sulphuric acid is used to strip the gold from the carbon; as a result, acid residues from this process reduce soil acidity.^42^

### Comparison of heavy metal concentrations with standard concentrations

There is currently no standard for heavy metals in soil in Myanmar, therefore the measured heavy metal concentrations in the present study were compared with the standard soil guidelines in Central and Eastern Europe adopted from the Food and Agriculture Organization of the United Nations and International Soil Reference Centre.^43^ In Central and Eastern Europe, these standard concentrations have been used to assess soil degradation caused by anthropogenic activities, including mining. These standards are categorized according to A-value, B-value, and C-value. Soil concentrations below the A-value indicate clean soil, those between A-value and B-value indicate slightly polluted soil, the range between B-value and C-value refers to moderately contaminated soil, and concentrations above C-value indicate strongly contaminated soil.^43^

Comaprison of the mean heavy metal concentrations in this study with the actual A, B and C values is shown in Table 2.

**Table 2:** Comparison of Heavy Metal Concentrations with Standard Concentrations (mg/kg)

**Heavy metals across gold mining stages**	**Gold mining stages by site**			**Standard concentrations across organizations/countries**						

				**Central and Eastern Europe**			**South Africa**	**China**	**Australia**	**Canada**

	**Site A**	**Site B**	**Site C**	A-value^*^	**B-value**	**C-value**				
Ore processing stage										
As	5.69	6.55	1.80	20.00	30.00	50.00	5.80	40.00	20.00	18.00
Cd	0.27	0.27	0.33	1.00	5.00	20.00	7.50	1.00	3.00	1.20
Hg	0.47	0.68	0.53	0.50	2.00	10.00	0.93	1.50	1.00	0.27
Pb	9.73	11.80	12.80	50.00	150.00	600.00	20.00	500.00	300.00	120.00
Sluicing stage										
As	1.04	9.02	1.31	20.00	30.00	50.00	5.80	40.00	20.00	18.00
Cd	0.36	0.40	0.13	1.00	5.00	20.00	7.50	1.00	3.00	1.20
Hg	0.15	0.35	0.51	0.50	2.00	10.00	0.93	1.50	1.00	0.27
Pb	8.60	10.27	7.67	50.00	150.00	600.00	20.00	500.00	300.00	120.00
Panning stage										
As	1.24	16.25	14.89	20.00	30.00	50.00	5.80	40.00	20.00	18.00
Cd	1.32	1.67	0.61	1.00	5.00	20.00	7.50	1.00	3.00	1.20
Hg	4.86	1.53	0.58	0.50	2.00	10.00	0.93	1.50	1.00	0.27
Pb	34.40	74.67	27.33	50.00	150.00	600.00	20.00	500.00	300.00	120.00
Amalgamation stage										
As	15.58	20.72	22.17	20.00	30.00	50.00	5.80	40.00	20.00	18.00
Cd	3.03	3.07	1.13	1.00	5.00	20.00	7.50	1.00	3.00	1.20
Hg	40.95	77.44	35.73	0.50	2.00	10.00	0.93	1.50	1.00	0.27
Pb	75.67	210.00	39.33	50.00	150.00	600.00	20.00	500.00	300.00	120.00

^*^ Soil concentrations below the A-value indicate clean soil, those between A-value and B-value indicate slightly polluted soil, the range between B-value and C-value refers to moderately contaminated soil, and concentrations above C-value indicate strongly contaminated soil.

In the present study, soil in the ore processing stage of all the mining sites was slightly contaminated (between A-value and B-value) with Hg. However, all the concentrations of heavy metals in the sluicing stage of all the gold mining sites were found to be clean (below A-value).

In the panning stage, Site A and Site B were slightly contaminated (between A-value and B-value) with Cd. Moreover, the results also showed moderately Hg-contaminated soil in Site A (between B-value and C-value), and slightly Hg-polluted soil in Site B and Site C (between A-value and B-value). In addition, slightly Pb-contaminated soil was found in Site B.

In the amalgamation stage, slightly As-polluted soil was found in Site B and Site C. Furthermore, the soil in all the mining sites was slightly polluted with Cd. Strongly Hg-contaminated soil was also found in the amalgamation stages of all the mining sites. In Site A, slightly Pb-polluted soil was found; whereas, moderately Pb-contaminated soil was found in this stage of Site B.

In addition to a comparison of heavy metal concentrations with the soil standard from Central and Eastern Europe, the heavy metal concentrations in this study were also compared with the standard concentrations from other high gold production countries, such as South Africa, China (Class III), Australia and Canada.^44^–^48^ The details of the comparison of heavy metal concentrations with the standard concentrations are shown in Table 2.

In the ore processing stage, the As concentration in Site B was greater than the standard concentration in South Africa; and the Hg concentration in Site A, Site B and Site C were slightly higher than the standard concentration in Canada. In the sluicing stage, the As concentration at Site B was higher than the standard concentration in South Africa; and Hg concentrations at Site B and Site C were slightly higher than standard concentration in Canada. In the panning stage, As concentrations at Site B and Site C were more than 2 times higher than the standard concentration in South Africa and Cd concentrations at Site A and Site B were slightly higher than the standard concentrations in China and Canada. The Pb concentrations at Site A and Site C surpassed the standard concentration in South Africa, and the Pb concentration at Site B was more than 3 times higher than the standard concentration in South Africa.

In the amalgamation stage, the As concentration at Site A was more than 2 times greater than the standard concentration in South Africa, and the As concentration at Site B and Site C were more than 3 times higher than the standard concentration in South Africa and slightly greater than the standard concentrations in Canada and Australia. The concentration of Cd at Site A and Site B was greater than the standard concentrations in China, Australia and Canada, while the Cd concentration at Site C surpassed the standard concentration in China. For Hg, all concentrations at Site A, Site B, and Site C were drastically higher than all of the standard concentrations.

For Pb concentrations in this stage, the concentration at Site A was more than 3 times higher than the standard concentration in South Africa; the concentration at Site B was extremely high and surpassed the standard concentration in South Africa and Canada; and the concentration at Site C was nearly 2 times greater than the standard concentration in South Africa.

Furthermore, comparing the heavy metal exposure dose with the reference dose and slope factor, the heavy metals in the soils of the present study showed concentrations high enough to affect human health via all three exposure routes and pose a cancer risk from directly contact with soils through oral, dermal and inhalation exposure *(Table 3).*

**Table 3:** Comparison of Heavy Metal Concentrations with Reference Dose and Cancer Slope Factor

**Heavy metals across gold mining stages**	**Concentration of heavy metals (mg/kg)**			**Non-cancer reference dose (mg/kg-day)**			**Cancer Slope factor (mg/kg/day)-l**			**Reference**

	**Site A**	**Site B**	**Site C**	**Oral**	**Dermal**	**Inhalation**	**Oral**	**Dermal**	**Inhalation**	
Ore processing stage										
As	5.690	6.550	1.800	0.0003	0.0003	0.0003	**1.5**	**1.5**	**15**	**44, 49,38,44**
Cd	0.270	0.270	0.330	0.0005	0.0005	0.000057	-	-	6.30	**44, 49**
Hg	0.470	0.680	0.530	0.0003	0.0003	0.000086	-	-	-	**44**
Pb	9.730	11.800	12.800	0.0036	0.0036	-	0.0085	-	0.042	**44, 50**
Sluicing stage										
As	1.040	9.020	1.310	0.0003	0.0003	0.0003	**1.5**	**1.5**	**15**	**44, 49**
Cd	0.360	0.400	0.130	0.0005	0.0005	0.000057	-	-	6.30	**44, 49**
Hg	0.150	0.350	0.510	0.0003	0.0003	0.000086	-	-	-	**44**
Pb	8.60	10.270	7.670	0.0036	0.0036	-	0.0085	-	0.042	**44, 50**
Panning stage										
As	1.240	16.250	14.890	0.0003	0.0003	0.0003	**1.5**	**1.5**	**15**	**44, 49**
Cd	1.320	1.670	0.610	0.0005	0.0005	0.000057	-	-	6.30	**44, 49**
Hg	4.860	1.530	0.580	0.0003	0.0003	0.000086	-	-	-	**44**
Pb	34.40	74.670	27.330	0.0036	0.0036	-	0.0085	-	0.042	**44, 50**
Amalgamation stage										
As	15.580	20.720	22.170	0.0003	0.0003	0.0003	**1.5**	**1.5**	**15**	**44, 49**
Cd	3.030	3.070	1.130	0.0005	0.0005	0.000057	-	-	6.30	**44, 49**
Hg	40.950	77.440	35.730	0.0003	0.0003	0.000086	-	-	-	**44**
Pb	75.670	210.000	39.330	0.0036	0.0036	-	0.0085	-	0.042	**44, 50**

## Conclusions

The present study revealed the soil in the study area to be contaminated with As, Cd, Hg and Pb. The highest concentrations of heavy metals were generally found in the amalgamation stages for all of the gold mining sites, although the heavy metal concentrations did not differ significantly between gold mining stages. Furthermore, the concentrations of heavy metals were greater than the standard concentrations from other countries. Due to a severe lack of personal protective equipment, small-scale gold miners in Myanmar are unprotected from occupational exposure to these metals. Miners are frequently exposed to heavy metals in the soil which pose a risk of cancer and other health risks. If miners do not wear proper personal protective equipment at work, they may experience adverse health effects due to long periods of exposure. Moreover, those living near gold mining areas could be exposed to heavy metals released from gold mining sites through inhalation, digestion, and dermal absorption. Therefore, a comprehensive health risk assessment for nearby residents and miners considering exposures to heavy metals should be conducted in future studies.
